# Incidental Chilaiditi Sign Identified During Evaluation for Symptomatic Cholelithiasis: Implications for Surgical Planning

**DOI:** 10.7759/cureus.113896

**Published:** 2026-08-03

**Authors:** Rafael Avilés Encarnación, Kennedy O'Neill, Julia L Armstrong, Allison Meihofer, Orlando Arce Renta

**Affiliations:** 1 Department of Internal Medicine, Larkin Community Hospital, Hialeah, USA; 2 Osteopathic Medical School, Nova Southeastern University Dr. Kiran C. Patel College of Osteopathic Medicine, Davie, USA; 3 Internal Medicine, VA Caribbean Healthcare System, San Juan, PRI

**Keywords:** chilaiditi sign, chilaiditi syndrome, cholelithiasis, laparoscopic cholecystectomy, radiographic findings

## Abstract

The Chilaiditi sign is a rare radiographic finding characterized by the interposition of the colon between the liver and the right hemidiaphragm. Although typically asymptomatic, it may complicate upper abdominal procedures or mimic pneumoperitoneum. We present a 73-year-old male with symptomatic cholelithiasis whose preoperative computed tomography (CT) imaging incidentally revealed the Chilaiditi sign. Given this finding, the standard umbilical approach for peritoneal access was avoided, and pneumoperitoneum was instead established via left upper quadrant (LUQ) Veress needle insertion to reduce the risk of injury to the interposed colon. Port placement was similarly adjusted away from the right upper quadrant. The procedure was completed without complications, and the patient was discharged on postoperative day four. This case highlights the importance of recognizing the Chilaiditi sign on preoperative imaging, as identification of this anatomic variant can directly influence peritoneal entry technique, trocar positioning, and overall operative safety during laparoscopic procedures.

## Introduction

The Chilaiditi sign is an uncommon radiographic finding in which a loop of bowel, most often the hepatic flexure or transverse colon, is interposed between the liver and the right hemidiaphragm [1]. It differs from Chilaiditi syndrome, where the same radiographic finding is accompanied by gastrointestinal symptoms, such as abdominal pain, nausea, or bowel obstruction [1].

The pathogenesis is thought to be multifactorial, involving hepatic factors (e.g., cirrhosis, hepatic atrophy, laxity of suspensory ligaments), intestinal factors (e.g., colonic redundancy, abnormal motility), and diaphragmatic factors (e.g., diaphragmatic thinning, elevated intrathoracic pressure) that collectively alter the normal spatial relationships among the liver, colon, and diaphragm [2,3].

Recognition is important because it can mimic free intraperitoneal air on imaging and may alter the operative field during upper abdominal surgery [4]. The reported prevalence ranges from 0.025% to 0.28% on plain radiographs and 1% to 2% on CT scans [4]. Detection has increased with the widespread use of cross-sectional imaging, and the finding is reported more often in older adults, with a male-to-female ratio of approximately 4:1 [5]. For surgeons, preoperative identification is particularly critical during laparoscopic procedures involving the upper abdomen, where the interposed bowel occupies the operative field and standard port placement may require modification to avoid iatrogenic injury.

Previous case reports have emphasized the clinical relevance of this finding, including diagnostic confusion with pneumoperitoneum prompting unnecessary surgical exploration, mimicry of a bile leak on hepatobiliary scintigraphy during evaluation for acute cholecystitis, and complications such as transverse colon volvulus and high-grade small-bowel obstruction requiring operative intervention [2,3,6,7,8]. A review of the available literature reveals no previously published cases specifically documenting the incidental preoperative identification of the Chilaiditi sign during workup for symptomatic cholelithiasis with subsequent modification of laparoscopic peritoneal entry technique and trocar placement. This case therefore represents a unique contribution to the surgical literature on the operative implications of this anatomic variant.

We present a case of an incidentally identified Chilaiditi sign during evaluation for symptomatic cholelithiasis, highlighting the importance of recognizing this anatomic variant before laparoscopic surgery to prevent bowel injury.

## Case presentation

A 73-year-old Caucasian male with a past medical history of gastroesophageal reflux disease (GERD), gastritis, and osteoarthritis presented with sudden epigastric and mid-abdominal pain that began two hours before arrival. The pain was non-radiating and was not associated with nausea, vomiting, diarrhea, constipation, fever, chills, weight loss, or chest pain. He reported regular bowel movements with the passage of flatulence and denied alcohol or tobacco use. He had no prior abdominal surgeries or endoscopic procedures.

On examination, the patient was hemodynamically stable with mild tachycardia and a blood pressure of 140/80 mmHg. Abdominal examination revealed mild epigastric tenderness without peritoneal signs. Laboratory studies demonstrated mild leukocytosis with a left shift, mild aspartate aminotransferase (AST) elevation (AST 54 U/L) with normal alanine aminotransferase (ALT) (ALT 29 U/L), and an elevated total bilirubin of 1.7 mg/dL, predominantly indirect. A summary of laboratory findings with reference ranges is provided in Table 1.

**Table 1:** Laboratory evaluation with reference ranges

Laboratory value	Admission	Day 2	Reference range
White blood cell count (WBC)	12.5 × 10³/µL	9.7 × 10³/µL	4.5–11.0 × 10³/µL
Neutrophil percentage	83.7%	81.2%	50–70%
Absolute neutrophil count (ANC)	10.48 × 10³/µL	7.85 × 10³/µL	1.8–7.7 × 10³/µL
Alanine aminotransferase (ALT)	29 U/L	508 U/L	7–56 U/L
Aspartate aminotransferase (AST)	54 U/L	560 U/L	10–40 U/L
Total bilirubin	1.7 mg/dL	3.7 mg/dL	0.1–1.2 mg/dL
Direct bilirubin	0.5 mg/dL	Not calculated	0.0–0.3 mg/dL

**Figure 1:**
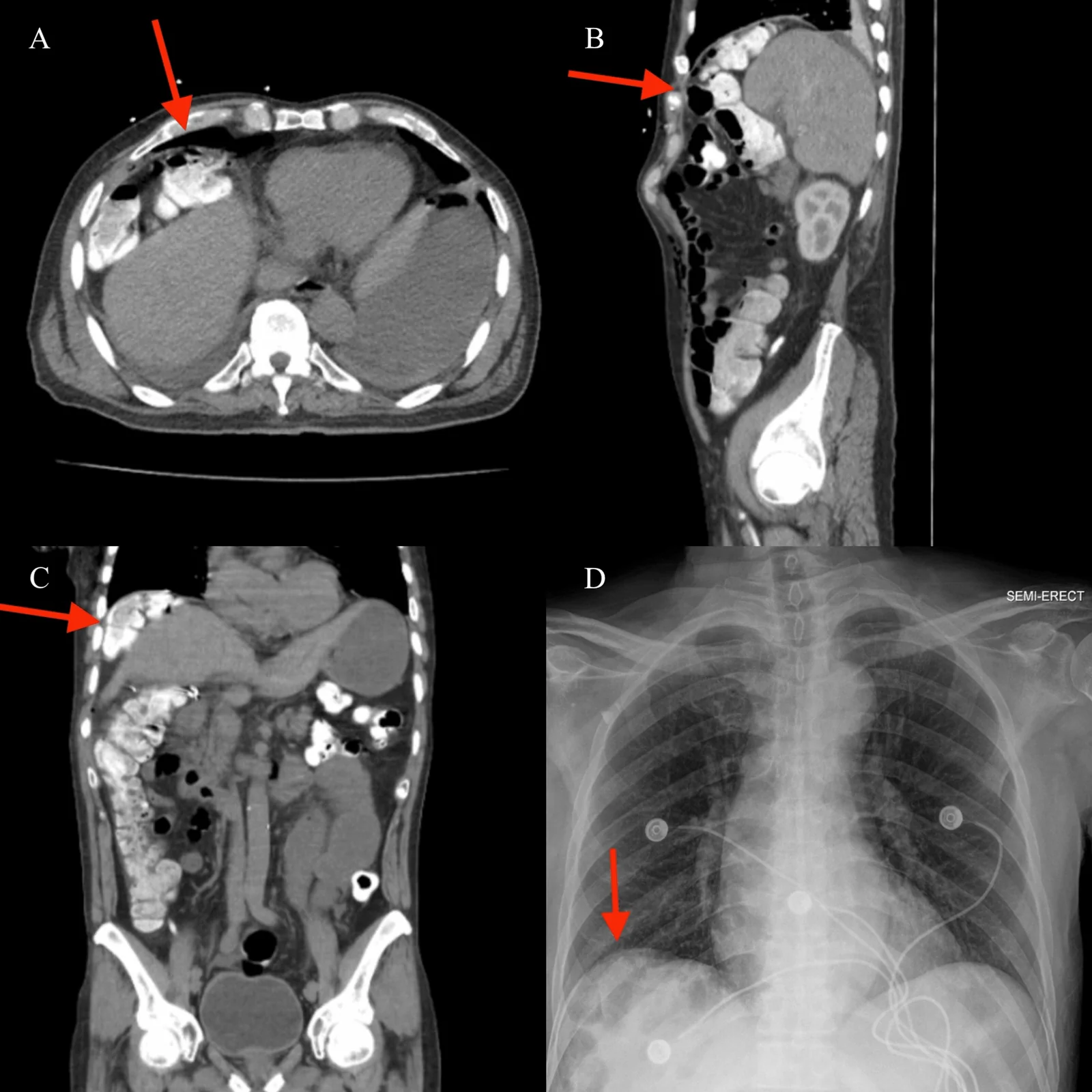
Imaging demonstrating the Chilaiditi sign. (A–C) Axial, sagittal, and coronal computed tomography images showing colonic interposition between the liver and right hemidiaphragm (red arrows). (D) Chest radiograph demonstrating subdiaphragmatic gas (red arrow) consistent with the Chilaiditi sign.

A right-upper-quadrant ultrasound demonstrated cholelithiasis with gallbladder sludge and no evidence of cholecystitis. To further evaluate the abdominal pain and exclude other intra-abdominal pathology, a contrast-enhanced CT scan of the abdomen and pelvis was obtained. Incidentally, CT imaging revealed interposition of a colonic loop between the liver and right hemidiaphragm, consistent with the Chilaiditi sign (Figure 1). No radiographic findings suggested bowel obstruction, pneumoperitoneum, or inflammatory changes that would suggest Chilaiditi syndrome.

Initial management included intravenous fluids, analgesics, antiemetics, and bowel rest. Because the patient had no symptoms attributable to the interposed bowel, the finding was classified as the Chilaiditi sign rather than Chilaiditi syndrome. General surgery was consulted for suspected biliary colic. On hospital day two, liver enzymes peaked (ALT 508 U/L, AST 560 U/L, total bilirubin 3.7 mg/dL). Magnetic resonance cholangiopancreatography (MRCP) confirmed gallstones and sludge without choledocholithiasis.

Given the preoperative identification of the Chilaiditi sign, the standard umbilical Veress needle entry was avoided due to the risk of injury to the interposed colon occupying the right upper quadrant. Pneumoperitoneum was instead established via Veress needle insertion in the left upper quadrant, and port placement was adjusted accordingly, with a 5 mm optic trocar placed above the umbilicus, two 5 mm trocars in the right subcostal area, and a 12 mm trocar in the epigastric area. This configuration intentionally displaced the operative field away from the interposed colonic segment. The surgery proceeded without complications; critical view of safety was achieved, the cystic duct and cystic artery were clipped and transected, and no common bile duct stones were identified on intraoperative cholangiogram. The gallbladder was removed via the 12 mm epigastric port. The patient tolerated the procedure well and was discharged on postoperative day four without complications.

## Discussion

**Figure 2:**
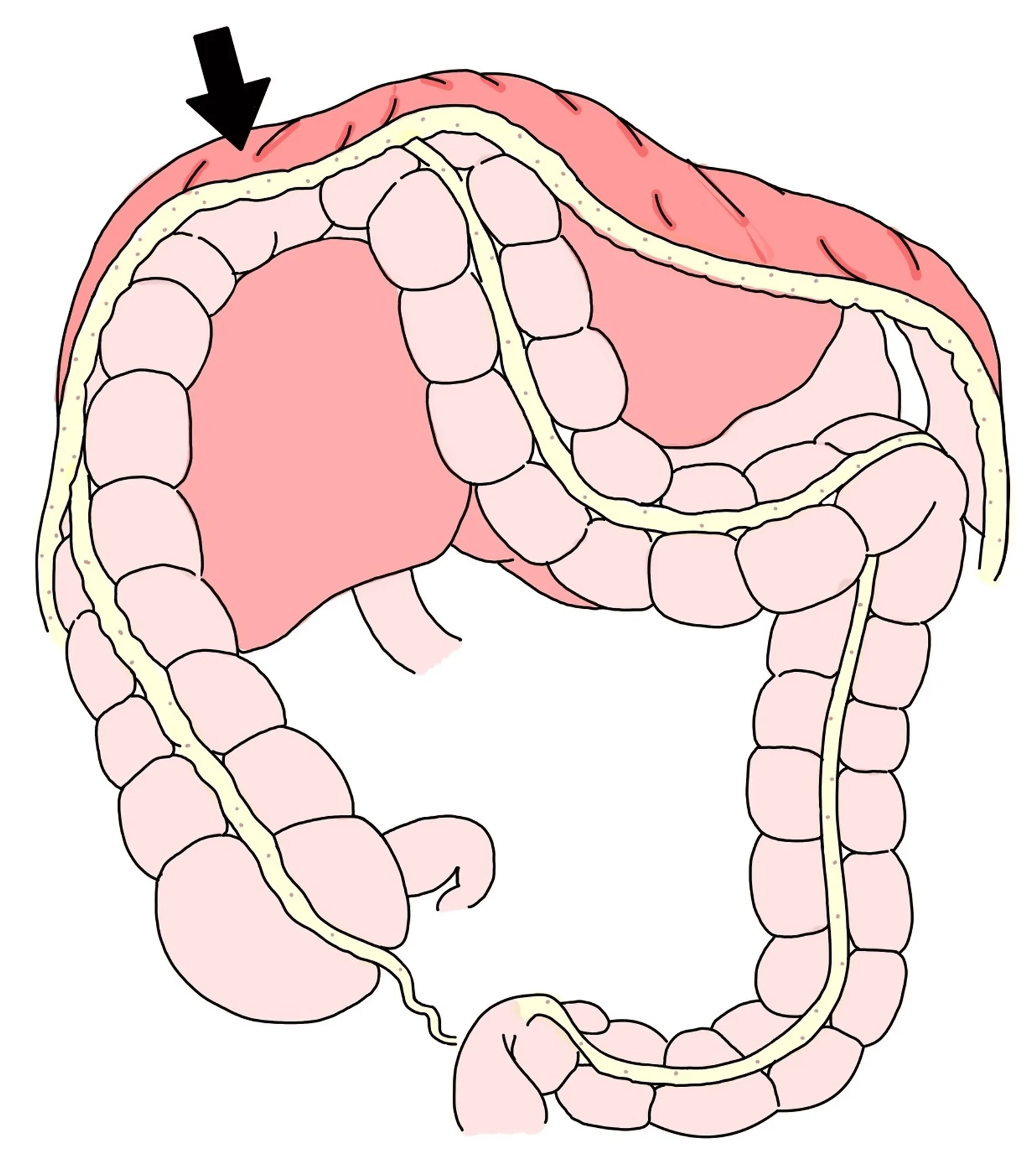
Chilaiditi sign Illustration showing the interposition (arrow) of the colon between the liver and the right hemidiaphragm. Illustration created by Allison Meihofer using GoodNotes (GoodNotes Limited, London, UK).

The Chilaiditi sign is characterized by the interposition of the colon between the liver and the right hemidiaphragm and is most often detected incidentally on imaging performed for unrelated indications (Figure 2) [1]. By contrast, Chilaiditi syndrome refers to the same anatomic configuration accompanied by symptoms such as abdominal pain, bowel obstruction, or volvulus [1]. Although the Chilaiditi sign is often detected incidentally, recognition of this variant is important for diagnostic interpretation and surgical planning [4].

Because the Chilaiditi sign is typically asymptomatic, no intervention is required, and management consists of recognition and documentation. However, accurate identification is important, as it may be mistaken for pneumoperitoneum on imaging, leading to unnecessary diagnostic procedures or surgical exploration [9]. Awareness is also essential when planning upper abdominal procedures because the interposed bowel increases the risk of iatrogenic injury [9].

In patients who develop Chilaiditi syndrome, initial management is typically conservative, including bowel rest, intravenous fluids, and decompression, with surgery reserved for complications, such as obstruction, ischemia, or failed conservative therapy [9].

A critical aspect of diagnosis is distinguishing the Chilaiditi sign from true pneumoperitoneum on imaging. On plain radiograph, the presence of haustral markings or a visible bowel wall within the subdiaphragmatic gas shadow favors the Chilaiditi sign over free intraperitoneal air. On CT, bowel interposition can be directly visualized with clear delineation of the bowel wall, haustra, and absence of free intraperitoneal air along the peritoneal margins or beneath the contralateral hemidiaphragm. These imaging features are important for clinicians to recognize across modalities, as misidentification of the Chilaiditi sign as pneumoperitoneum may prompt unnecessary and potentially harmful surgical intervention [8].

Several anatomic and physiologic factors can contribute to the development of the Chilaiditi sign by altering the normal spatial relationships among the liver, colon, and diaphragm [10]. Reported contributors include hepatic factors such as cirrhosis, hepatic atrophy, ascites, or laxity of the hepatic suspensory ligaments; intestinal factors such as colonic redundancy, megacolon, or abnormal colonic motility; and diaphragmatic factors such as diaphragmatic thinning or conditions that alter intrathoracic pressure, including chronic obstructive pulmonary disease (COPD). Additional associations include chronic constipation, prior abdominal surgery, obesity, and aerophagia [10].

The surgical implications of the Chilaiditi sign are particularly relevant in the context of laparoscopic cholecystectomy. The standard four-port technique employs a supraumbilical camera port, a subxiphoid epigastric port for dissection, and two right upper quadrant working ports in the midclavicular and anterior axillary lines, with pneumoperitoneum established via umbilical Veress needle insertion [9,11]. In the presence of colonic interposition in the right upper quadrant, this standard configuration poses a significant risk of inadvertent bowel injury during peritoneal access and trocar placement. Failure to recognize this anatomy preoperatively may result in iatrogenic injury during trocar insertion, peritoneal entry, or dissection, particularly in laparoscopic cholecystectomy, hepatic resections, or diaphragmatic repairs [9,11].

In the present case, preoperative recognition of the Chilaiditi sign directly informed both the entry technique and port configuration. LUQ Veress needle insertion was selected over the standard umbilical approach to avoid the interposed colonic segment occupying the right upper quadrant, and port placement was adjusted with a 5 mm optic trocar above the umbilicus, two 5 mm trocars in the right subcostal area, and a 12 mm trocar in the epigastric area, intentionally displacing the operative field away from the interposed bowel. This approach is consistent with established principles of safe laparoscopic entry when right upper quadrant anatomy is distorted [9,11].

The previously cited cases illustrate the varied clinical presentations of the Chilaiditi sign and syndrome. Fiumecaldo and Buck described a case requiring exploratory laparotomy for high-grade small bowel obstruction, representing the most severe end of the clinical spectrum [2]. Xu et al. reported a presentation with sudden chest pain and dyspnea, demonstrating that the condition can mimic cardiopulmonary pathology [3]. Weng et al. described a rare left-sided variant with transverse colon volvulus requiring operative intervention [6]. Murphy et al. identified obesity as a contributing factor, which was not present in our patient [12]. Ben Ismail et al. reported a case where the Chilaiditi sign was initially mistaken for pneumoperitoneum, underscoring the diagnostic confusion this finding can generate [8]. In contrast to these cases, which largely involved symptomatic presentations or diagnostic errors, our patient's Chilaiditi sign was identified incidentally on preoperative CT during workup for symptomatic cholelithiasis. Critically, the finding was recognized prospectively and used to modify operative planning before any intervention, thereby preventing a potential complication rather than responding to one.

## Conclusions

The Chilaiditi sign is an uncommon radiographic finding characterized by the interposition of a segment of the colon between the liver and the right hemidiaphragm and is most often identified incidentally on imaging. Although typically asymptomatic, recognition of this anatomic variant is important because it may mimic pneumoperitoneum and influence the approach to abdominal procedures. In the present case, preoperative identification of the Chilaiditi sign during evaluation for symptomatic cholelithiasis prompted modification of the operative approach, including avoidance of standard umbilical peritoneal entry, LUQ Veress needle insertion, and adjusted trocar positioning to displace the operative field away from the interposed colon, facilitating safe laparoscopic cholecystectomy without iatrogenic bowel injury. Surgeons encountering the Chilaiditi sign on preoperative imaging should consider alternative peritoneal entry sites and modified port placement to reduce the risk of iatrogenic bowel injury during laparoscopic procedures.
